# Prostate Cancer Metastatic to the Peritoneum: A Road Less Traveled by a Common Malignancy

**DOI:** 10.7759/cureus.4222

**Published:** 2019-03-11

**Authors:** Serene A Tareen, Joshua Rodriguez, Phillis Wu

**Affiliations:** 1 Internal Medicine, Olive View - University of California Los Angeles (UCLA) Medical Center, Los Angeles, USA; 2 Hematology & Oncology, Olive View - University of California Los Angeles (UCLA) Medical Center, Los Angeles , USA; 3 Hematology & Oncology, Olive View - University of California Los Angeles (UCLA) Medical Center, Los Angeles, USA

**Keywords:** prostate cancer, metastatic, peritoneum, omentum, peritoneal carcinomatosis, bone metastases, signet ring carcinoma, peritoneal tuberculosis, india and tuberculosis, psa

## Abstract

A 70-year-old Indian male with a history of a Gleason 7 (3+4) prostate cancer presented with abdominal ascites. Imaging was remarkable for peritoneal carcinomatosis as well as possible metastases to the bladder and seminal vesicle. Given the atypical pattern of presentation, further investigation was performed with studies of the ascites fluid. Cytology from the ascites fluid returned consistent with malignant cells of prostatic origin. His treatment course included androgen deprivation therapy (ADT), docetaxel, abiraterone, and cabazitaxel. He had eventual progression and worsening of his disease and performance status and was transitioned to hospice. This case demonstrated the importance of pursuing a thorough diagnostic evaluation, when faced with a rare presentation of a common malignancy. Furthermore, it illustrated the challenges incurred when tailoring standard regimens to best address the needs of the whole patient and not simply their disease.

## Introduction

The common metastatic sites for prostate cancer are bone, lymph nodes, liver, and thorax with the vast majority of cases presenting with osteoblastic lesions involving the axial skeleton [1]. It is exceedingly rare for prostate cancer to metastasize to the peritoneum, especially in the absence of osseous metastases. We present a rare case of metastatic prostate cancer to the omentum without evidence of bony metastases.

## Case presentation

**Figure 1:**
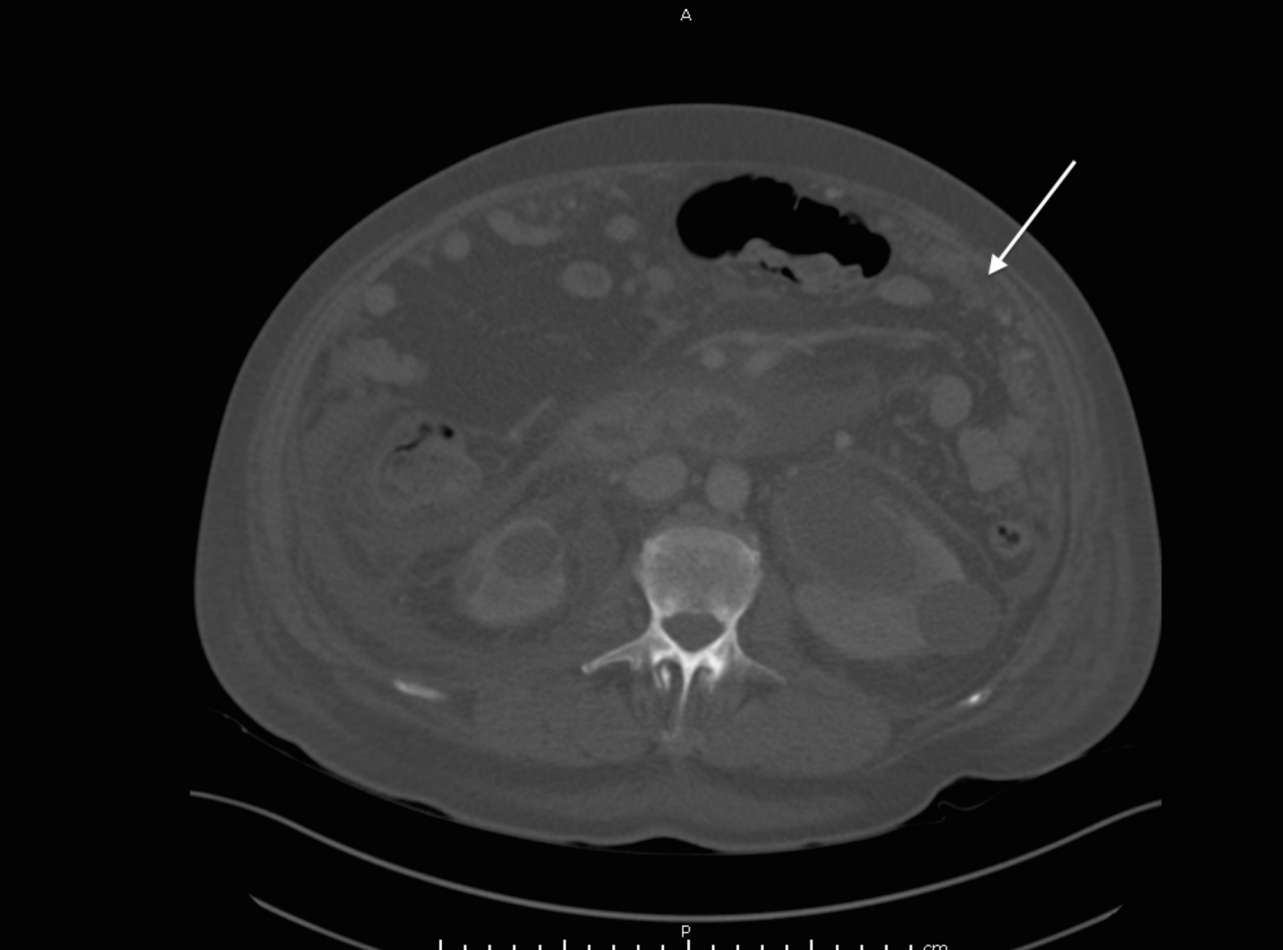
Peritoneal carcinomatosis CT abdomen and pelvis scan performed without intravenous contrast demonstrating peritoneal carcinomatosis CT: computed tomography

A 70-year-old Indian male with a history of a Gleason 7 (3+4) prostate cancer who underwent aborted prostatectomy a week prior, presented with weakness, abdominal distention, decreased urine output, increased lower extremity edema, and constipation for five days. On exam, his vital signs were normal. His abdomen was soft but distended, and bilateral lower extremities showed pitting edema to the level of the knees. Initial laboratory studies were notable for sodium of 117 mmol/L, potassium of 6.3 mmol/L, creatinine of 9.92 mg/dL, and white blood cell count of 21 x 10^8^/L. Computed tomography (CT) of the abdomen and pelvis was remarkable for diffuse urinary bladder wall thickening extending to the level of the seminal vesicles, bilateral hydronephrosis, diffuse peritoneal carcinomatosis with moderate ascites, and small low-density lesions in the right lower lobe of the liver too small to characterize (Figure 1). The patient subsequently underwent emergent hemodialysis followed by interventional radiology guided nephrostomy tube placement. Nuclear medicine (NM) bone scan did not show definitive evidence of osseous metastases.

**Figure 2:**
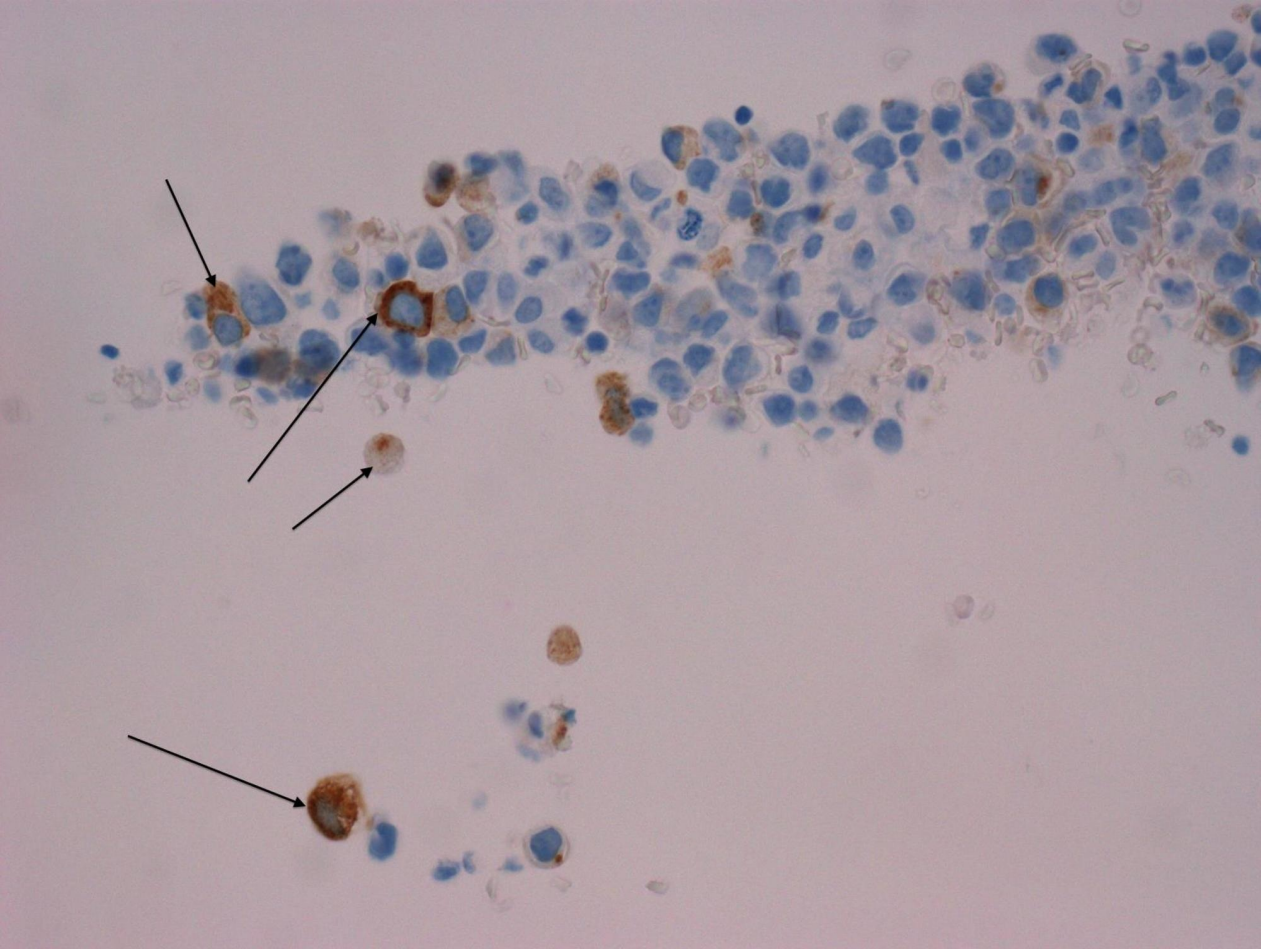
PSA immunohistochemistry stain PSA: prostate-specific antigen

**Figure 3:**
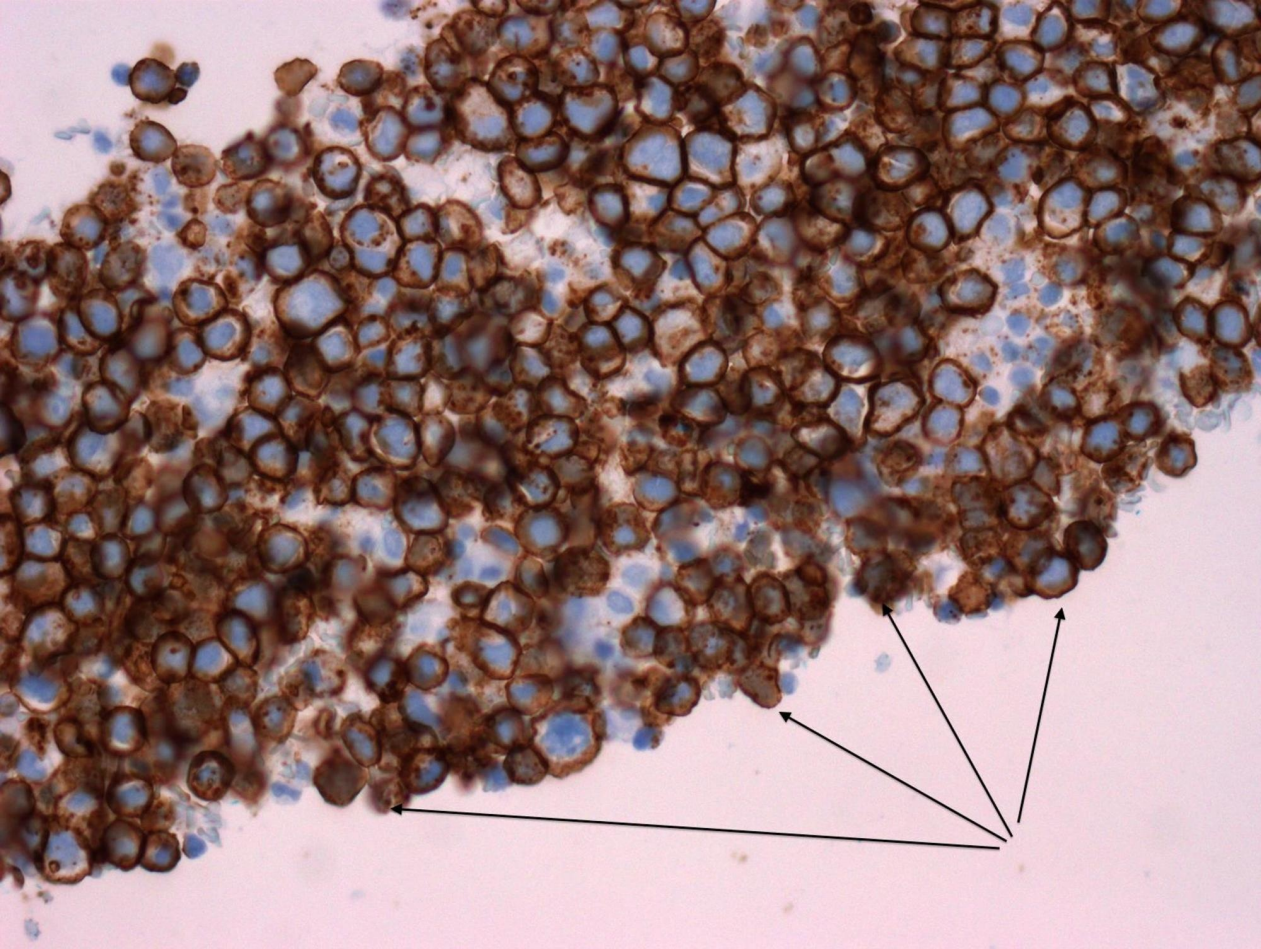
Immunohistochemistry stain positive for monoclonal antibody Ber-EP4

**Figure 4:**
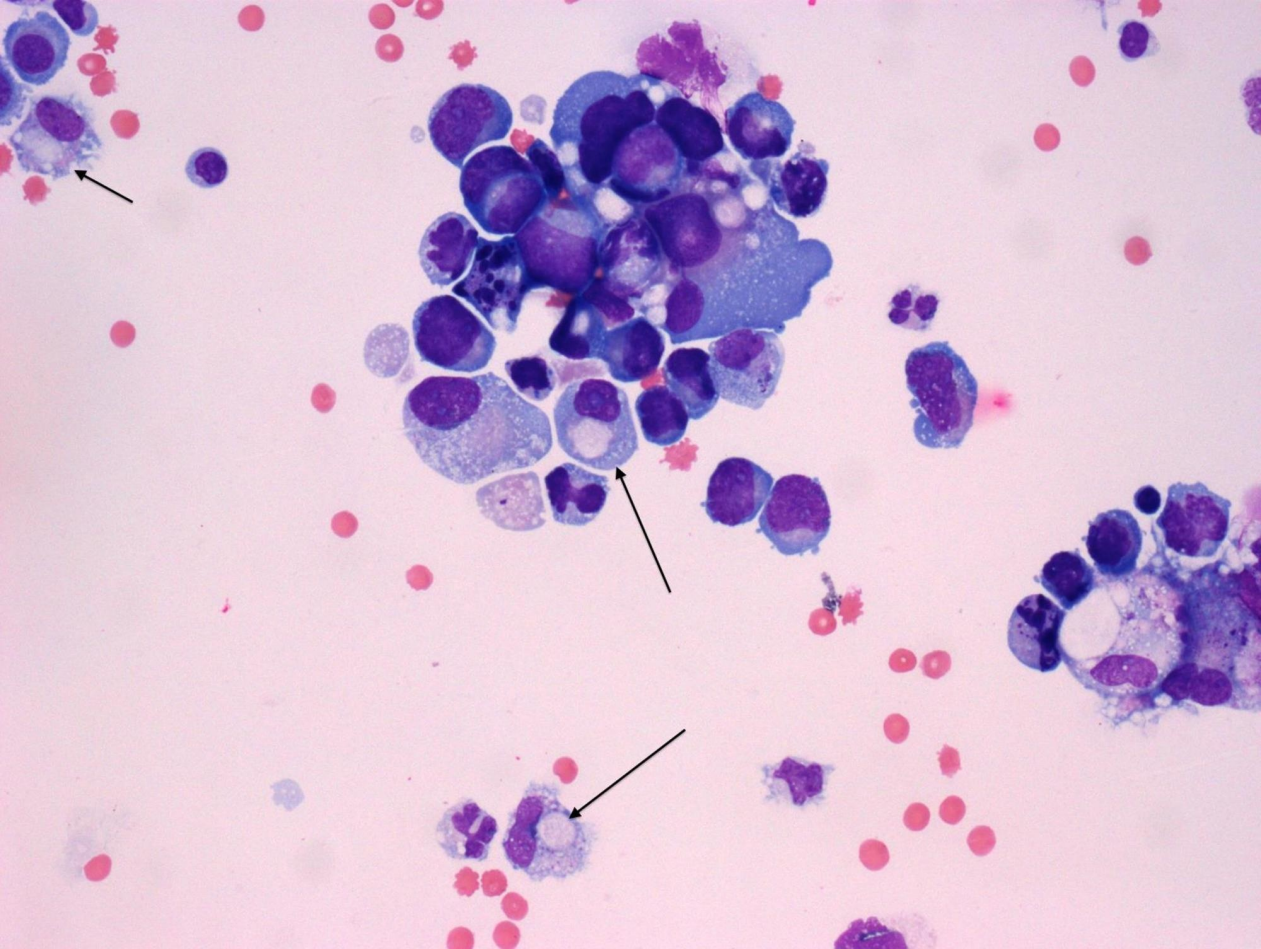
H&E stain with signet ring cell morphology The above H&E stain shows markedly atypical cells with oval to pleomorphic nuclei, high nucleus to cytoplasm ratios, prominent nucleoli, and a moderate amount of basophilic cytoplasm. Some cells (marked with arrows) appear dis-cohesive, with eccentrically located nuclei, resembling signet ring cell morphology. H&E: hematoxylin and eosin

Given the rarity of peritoneal involvement of prostate cancer, the pursuit of confirmatory testing was necessary to evaluate for other primary cancers. In addition, the patient’s origin from a tuberculosis endemic region raised concern for possible peritoneal tuberculosis. Ascitic fluid cytology studies confirmed the presence of malignant cells. Immunohistochemistry staining positive for prostate-specific antigen (PSA) and Ber-EP4 (a monoclonal antibody used to distinguish between adenocarcinoma and reactive mesothelium) was compatible with primary prostate adenocarcinoma (Figures 2-3) [2]. However, the hematoxylin and eosin (H&E) staining also showed focal signet-ring cell features concerning for possible gastrointestinal carcinoma primary (Figure 4). Ascitic fluid cultures for acid-fast bacilli, *Mycobacterium tuberculosis* polymerase chain reaction (PCR) and Quantiferon assays, were negative. The patient declined further tissue analysis via cystoscopy.

**Figure 5:**
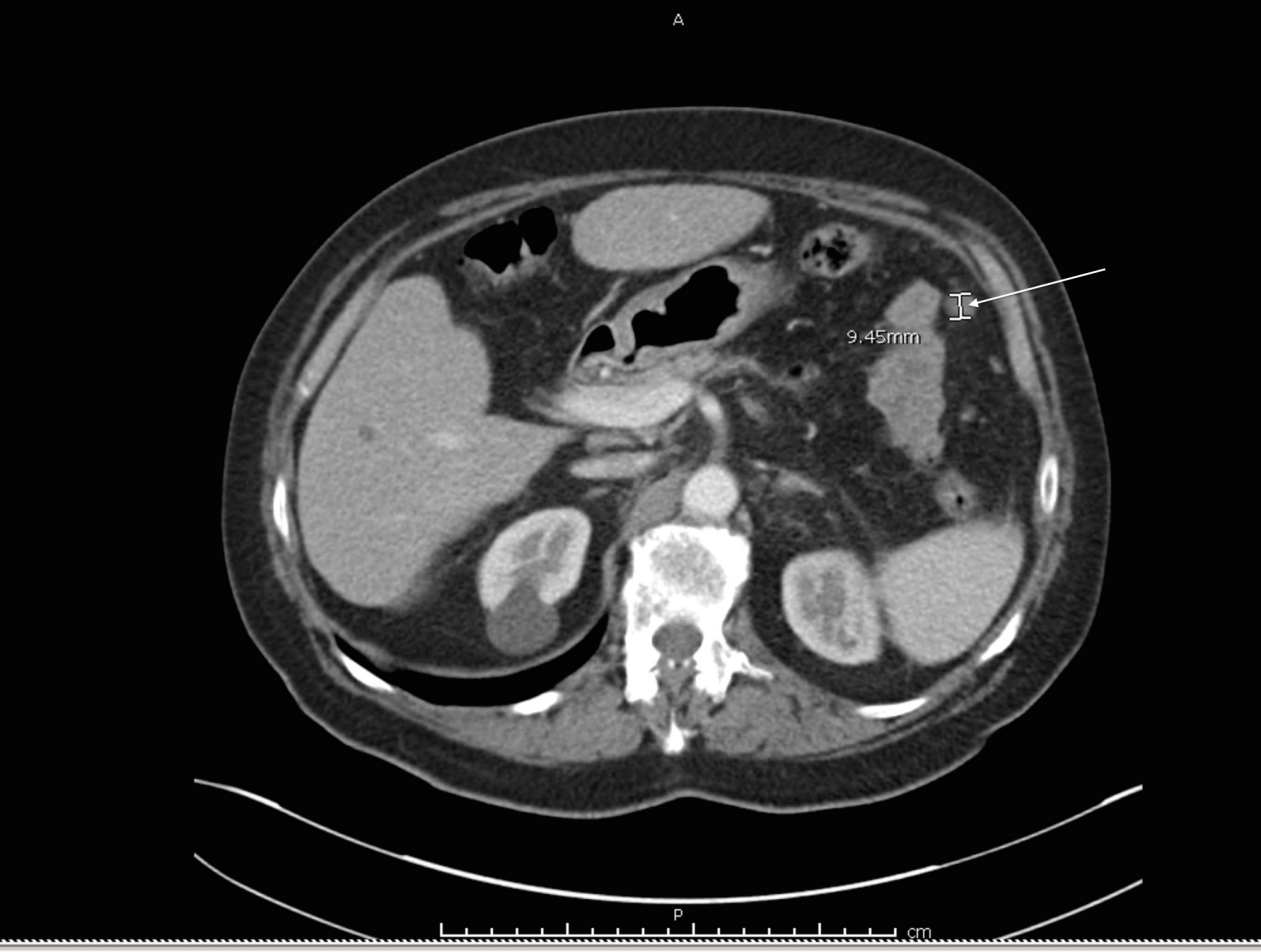
Large peritoneal nodule CT scan study was done with intravenous contrast five months after presentation highlighting an enlarging peritoneal nodule. CT: computed tomography

The patient was treated with androgen deprivation therapy (ADT), which included leuprolide and bicalutamide. Extensive discussions were held with regard to the addition of docetaxel to ADT according to the chemo-hormonal therapy vs. androgen ablation randomized trial for extensive disease in prostate cancer (CHAARTED) [3], but the patient declined as he felt too weak. The patient’s total serum PSA decreased from 38.2 ng/ml to 4.7 ng/ml over four months. Repeat staging CT scan of the abdomen and pelvis that was done five months after the initiation of therapy showed an overall improvement in the peritoneal carcinomatosis. However, there was an increase in the size of some nodules in the omentum as well as a new soft tissue mass extending into the seminal vesicle consistent with progressive disease (Figure 5). NM bone scan was negative for osseous metastases and patient’s serum PSA was 192.

The patient was deemed castrate-resistant with plans to start on abiraterone and prednisone therapy. Before the patient could start therapy, he developed hematuria, requiring admission to the hospital for bladder outlet obstruction from compressive effects of the mass. Repeat CT scans showed increased tumor burden including the progression of the peritoneal carcinomatosis. Although the patient was out of the window of benefit per the CHAARTED trial, he was started on docetaxel given his high volume disease while continuing his Lupron. After cycle six of docetaxel, his total serum PSA down-trended to 3.38 ng/ml with repeat CT imaging showing the response in almost all target lesions, except for an increase in the size of a nodule at the vesicoureteral junction. Given the concern for progression, the patient was subsequently started on abiraterone and prednisone. After six months of therapy, the patient had progression with worsening peritoneal carcinomatosis and a noted increase in his PSA to 4.66 ng/ml. After discussion with the patient regarding his goals for treatment, it was decided to start therapy with cabazitaxel. After one cycle of therapy, the patient had improvement of his symptoms with a decrease in his PSA. Upon completion of his fourth cycle, however, the patient developed sepsis, requiring admission to the hospital. Given his worsened functional status, the patient was deemed an unsuitable candidate for further chemotherapy and was later transitioned to home hospice. 

## Discussion

In a population-based analysis of 75,000 patients with metastatic prostate cancer, the most frequent sites of metastases for prostate cancer were bone (84%), distant lymph nodes (10.6%), liver (10.2%), and thorax (9.1%) [1]. At present, only a few cases of peritoneal carcinomatosis from metastatic prostate cancer have been reported. It is well known that variation in sites of metastases among cancer types exists, and these are reflective of inherent differences in cancer cell biology. This is exemplified by the unique propensity of prostate adenocarcinoma to spread to bone. The mechanism for metastases of prostate cancer is complex and not completely understood, but the pathophysiologic spread to bone has been studied extensively. A number of receptors expressed by prostate cancer cells mediating their spread to bone have been identified including chemokine receptor type four (CXCR4), chemokine receptor type seven (CXCR7), annexin II, integrin αvβ3, and Notch-Jagged [4]. As would be expected with such few cases reported, the mechanism of metastases of prostate cancer to the omentum has not been well described. One case report by Sheng et al. postulated port-site metastases after laparoscopic prostatectomy in a patient with peritoneal carcinomatosis [5]. An initial omental nodule proximal to the prostatectomy exit port site, two negative measurements of circulating tumor cells, and absence of other distant metastases supported this theory [5]. This hypothetical mechanism of spread has been questioned given the lack of evidence to support this. Additionally, the widespread use of robotic prostatectomy has not confirmed this finding. In the case of our patient, his diffuse peritoneal carcinomatosis was discovered only six days after his aborted prostatectomy and peritoneal involvement of his cancer was noted on initial staging imaging prior to the procedure. Although measurements of circulating tumor cells were not obtained in this case, we hypothesize that the spread of his cancer was hematogenous or through the lymphatic circulation to the omentum. Hematogenous spread in the absence of osseous metastasis would be very atypical however.

Another unique finding, in this case, was the histologic morphology of signet ring cells in the ascitic fluid. Signet-ring cell carcinoma (SRCC) is a rare form of cancer that carries a poor prognosis and is most commonly found in the stomach and colon [6-7]. Other less common primary sites of SRCC are the breast, pancreas, bladder, thyroid, and the lungs [6-7]. Primary SRCC of the prostate is even more rare, with roughly 60 cases reported in the literature [7]. In our patient with peritoneal carcinomatosis and rectal adhesions, a primary gastrointestinal malignancy was considered. His immunohistochemistry staining was positive for PSA and Ber-EP4 making SRCC of prostatic origin the most likely case. However, given there were no standardized treatment modalities for primary SRCC of the prostate, we pursued standardized treatment for prostate adenocarcinoma [7].

A common challenge encountered in the diagnosis of malignancy is the possibility of synchronous infections with similar presentations. Symptoms of tuberculosis in particular including fatigue, weight loss, fevers, and night sweats can mimic malignant processes. In this case of a patient from India with new abdominal ascites, a calcified mediastinal lymph node, and pulmonary granuloma, it was prudent to evaluate for peritoneal tuberculosis, as this would greatly influence management. According to the World Health Organization (WH0), India is on the list of 30 countries with the highest burden of tuberculosis. In 2017, India was one among eight countries that accounted for two-thirds of the world’s new tuberculosis cases [8]. Peritoneal tuberculosis is exceptionally rare and accounts for only 0.1-0.7% of tuberculosis cases [9]. There are several studies that can aid in the diagnosis of tuberculosis including acid-fast bacilli cultures, QuantiFERON assays, and PCR assays that vary in their degree of sensitivity and specificity [10]. The aforementioned results of these studies were negative in this case, making tuberculosis much less likely.

## Conclusions

This case demonstrates the importance of pursuing a thorough diagnostic evaluation, when faced with a rare presentation of a common malignancy. Additionally, the increased infection risk in the setting of malignancy, the difficulty in distinguishing between these processes, and the vast number of treatment approaches are demonstrative of the critical importance of considering infection in patients with the preexisting malignancies.
